# Biomechanical and clinical differences in muscle tone, stiffness, range of motion, and pain perception in children with cerebral palsy: a cross-sectional study

**DOI:** 10.3389/fphys.2025.1588084

**Published:** 2025-04-15

**Authors:** Ramón González-Matilla, Vanesa Abuín-Porras, Isabel Mínguez-Esteban, Alberto M. Heredia-Rizo

**Affiliations:** ^1^ Departamento de Fisioterapia, Investigación y Deporte. Centro Universitario FISIDEC, Universidad de Córdoba, Córdoba, Spain; ^2^ Centro de Atención Infantil Temprana, Universidad de Córdoba, Córdoba, Spain; ^3^ CTS 1110: Understanding Movement and Self in health from Science (UMSS) Research Group, Andalusia, Spain; ^4^ Department of Physiotherapy, Universidad Europea de Madrid, Faculty of Medicine, Health and Sports, Villaviciosa de Odón, Spain; ^5^ Instituto de Biomedicina de Sevilla, IBiS (Hospitales Universitarios Virgen del Rocío y Macarena/CSIC/Universidad de Sevilla), Sevilla, Spain; ^6^ Departamento de Fisioterapia, Universidad de Sevilla, Sevilla, Spain

**Keywords:** cerebral palsy, muscle stiffness, spasticity, pain perception, myotonometry

## Abstract

**Introduction:**

Spasticity and altered muscle tone are key features in children with neurodevelopmental disorders, particularly cerebral palsy (CP). They impact movement, range of motion (ROM), and pain perception, influencing functional abilities and quality of life. Understanding the intrinsic muscle differences in children with CP can help improve clinical assessment and therapeutic interventions. This study aims to evaluate differences in muscle tone, stiffness, ROM, and pain perception between children with CP and typically developing peers using objective biomechanical measures.

**Methods:**

An observational, cross-sectional study was conducted with 40 participants of both sexes (20 children with CP, 20 typically developing peers). Muscle tone and stiffness of the lower limb muscles were measured using the Myoton PRO device. ROM was assessed by goniometry, and pain perception was evaluated using the Visual Analog Scale during a Straight Leg Raise (SLR) test. A generalized linear mixed model was used to detect differences in myotonometry, ROM, and pain perception measurements. In participants with CP, the Pearson product-moment correlation coefficient analysis was used to explore possible associations between clinical features and muscle tone and stiffness.

**Results:**

Children with CP exhibited reduced ROM, with a significant group effect for hip flexion (P < 0.001; η^2^ = 0.843), knee extension (P < 0.001; η^2^ = 0.355), and ankle flexion (P < 0.001; η^2^ = 0.959) and higher pain perception during the SLR test (P < 0.001; η^2^ = 0.831), compared to controls. Myotonometry revealed significantly increased muscle stiffness of the rectus femoris (P = 0.004; η^2^ = 0.112) and adductor muscles (P = 0.019; η2 = 0.074) in the CP group, with no differences in muscle tone between the groups. Sex-related differences were found for muscle tone and stiffness, with males showing higher values. Correlation analyses indicated that adductor muscles stiffness was associated with CP severity.

**Conclusion:**

Children with CP demonstrate significant changes in ROM, pain perception, and muscle stiffness, emphasizing the need for targeted therapeutic interventions. These findings support the use of objective biomechanical tools for assessing muscle properties in clinical settings, contributing to better management strategies for spasticity-related impairments.

## Introduction

Muscle tone is a complex and dynamic state, resulting from hierarchical and reciprocal anatomical connectivity. It is a multidimensional concept regulated by input and output systems and has critical interplay with power and task performance requirements (Hidler and Rymer, 1999) that involve various body organs and systems, including bones, balance, and other neurological inputs, such as cardiovascular aspects and motivation (Beaudart et al., 2019). Atypical muscle tone is one of the most common clinical features in children with neurodevelopmental disorders, for example, in those with early brain injury or cerebral palsy (CP) (Goo et al., 2018).

Spasticity was first described as a “motor disorder characterized by a velocity-dependent increase in muscle tone, with exaggerated tendon jerks, resulting from hyperexcitability of the stretch reflex” (Trompetto et al., 2014). This definition emphasizes the fact that spasticity is just one component of the upper motoneuron syndrome. Currently, it has been refined to state that spasticity is not only velocity-dependent but also a length-dependent and acceleration phenomenon (Nourizadeh et al., 2024). Spasticity is often present in pathologies that affect the first motoneuron or directly impact the cerebral cortex (Trompetto et al., 2014). It is estimated that the annual incidence of lower limb spasticity ranges between 30 and 485 per 100,000 for stroke, and between 100 and 235 per 100,000 for traumatic brain injury (Bhimani and Anderson, 2014), with a prevalence of 2.1 per 1,000 for CP (Goo et al., 2018).

Spasticity not only disrupts the balance between muscle excitation and inhibition, but it can also result in secondary impairments, such as contractures (Lackritz et al., 2021), bone deformities (Handsfield et al., 2022), and pain (Penner et al., 2013). These complications may limit physical function and can also contribute to emotional distress, social isolation, and a decreased overall quality of life (Milinis and Young, 2016; Vural et al., 2020). The intrinsic differences that spastic muscles present compared to healthy muscles, such as the level of actin and myosin contraction and the number of sarcomeres affected, support what has been previously described regarding spasticity (Lieber and Fridén, 2019).

Although traditional clinical assessments of muscle tone and spasticity, such as the Modified Ashworth and Tardieu scales, are widely used, they have inherent limitations, including subjective interpretation, limited sensitivity, and low inter-examiner reliability (Yam and Leung, 2006) These drawbacks highlight the need for more precise evaluations. The present study seeks to address these limitations by utilizing objective tools, such as myotonometry, to quantify intrinsic muscle properties objectively.

Despite the expected differences in muscle mechanical and viscoelastic properties between children with neurological disorders and typically developing peers, this aspect has not yet been investigated in detail. This article aims to explore the clinical differences between spastic and normal muscles regarding muscle tone and stiffness, range of motion, and pain perception, and the potential implications these differences may have on movement, psychometric outcomes, and quality of life.

## Methods

### Study design

A cross-sectional, observational study was conducted, including children diagnosed with CP and age- and sex-matched healthy participants from the same population-based cohort. The study protocol received ethical approval from the Biomedical Research Ethics Committee of the Andalusian Government, Spain (approval code: CI 0214-N-20) and the Ethics Committee for Research with Medicines of the Hospital Clínico San Carlos, Madrid (approval code: 24/544-E). The study adhered to the STROBE guidelines and was prospectively registered in the OSF Registries (https://osf.io/yg7ax). Prior to enrollment, written informed consent was gained from all parents or legal guardians, along with informed assent from the participating minors. Participants and their families received detailed information about the study procedures and the potential risks or adverse effects. All study data was securely stored, ensuring confidentiality and anonymity.

### Population and setting

Children and adolescents, aged 6–17 years, diagnosed with CP, were recruited from various public and private specialized Early Intervention Centers in Southern Spain. The inclusion criteria were as follows: a) Gross Motor Function Classification System (GMFCS) level between I to IV, indicating preserved, but potentially severely limited, walking ability, with or without assistive devices (Rosenbaum et al., 2008). At level V, children and adolescents exhibit significant functional limitations, pronounced contractures, and severe positioning challenges, often requiring physical assistance and assistive technology to optimize head alignment, seating, standing, and mobility. All these factors may confound outcome measures, potentially compromising the accuracy and interpretability of the findings; b) increased muscle tone in the lower extremities, defined as a score greater than 0 on the Modified Tardieu Scale (Shu et al., 2021) and a positive response to the Duncan Ely Test (Lee et al., 2015); c) ability to communicate pain (Barney et al., 2018); and d) capacity to follow simple instructions and commands. Exclusion criteria included: a) previous lower limb surgery or interventions limiting joint mobility within the functional range (e.g., ankle joint arthrodesis); b) significant medication adjustments during the study that could influence muscle tone, whether increasing or decreasing it; c) uncontrolled epileptic seizures despite medication; d) having received botulinum toxin injections for the lower extremities within the 3 months before the study; and e) a concurrent new physical therapy treatment that could interfere with the study protocol.

The control group included healthy children and adolescents with normal sensorimotor development, no spasticity, and no previous history of neurological disorders. The same exclusion criteria applied to this group.

### Study protocol

Demographic and clinical data, including CP type, affected side, GMFCS level, and use of antispastic medication, were initially collected by the same examiners. Participants were allocated to the study groups depending on their medical diagnosis: CP or normal sensorimotor development. The evaluator remained blinded to the aims of the study and to the clinical condition of participants. All evaluations were conducted during a single session lasting approximately 30–35 min.

### Outcome measures

The primary outcome included muscle tone and stiffness of lower limb muscles, assessed using the Myoton PRO (Seo et al., 2023; Trybulski et al., 2024) (Myoton AS, Tallinn, Estonia), which has proven valid and reliable in individuals with neurological disorders, with a good to excellent intra- and inter-rater reliability to assess upper and lower extremity muscles in children with developmental disabilities, including CP (García-Bernal et al., 2021; Kutlutürk Yıkılmaz et al., 2024). With participants in a comfortable and relaxed supine or prone position, measurements were taken at two separate locations (proximal and distal) on the muscle bellies of the hamstrings (biceps femoris), quadriceps (rectus femoris), and adductor muscles (Bell, 2014; Agoriwo et al., 2022). Anatomical landmarks were identified using a wax pencil and the mean of two consecutive measurements, with a 30-s rest interval, was used for analysis.

For the biceps femoris, the proximal point was located midway between the ischial tuberosity and the head of the fibula, with the distal point positioned 6 cm caudally. For the rectus femoris, the proximal point was in the upper third of the muscle, between the anterior superior iliac spine and the upper pole of the patella, whereas the distal spot was located in the medial third of the muscle. For the adductor magnus, the proximal point was positioned in the upper third of the line extending from the internal femoral condyle and the ischiopubic ramus, while the distal point was in the medial third of the muscle (Agyapong-Badu et al., 2013; Drenth et al., 2018; García-Rueda et al., 2023).

Secondary outcomes comprised range of motion (ROM) measurements of the lower limb joints (Herrero et al., 2011; Johansen et al., 2020), and pain perception during the straight leg-raise test (SLR) (Marsico et al., 2016b). For ankle dorsiflexion, participants were seated comfortably at the edge of the table with their back supported and both lower limbs at rest. The goniometer (Enraf Nonius, PRIM group, Spain) was placed with the axis at the external malleolus, with one arm following the fifth metatarsal and the other the diaphysis of the fibula. For knee flexion, participants were in supine. The goniometer axis was at the lateral knee condyle, one arm aligned with the diaphysis of the femur and the other with the diaphysis of the fibula. For hip flexion, participants maintained the same supine position. The goniometer axis was placed at the greater trochanter of the femur, with one arm aligned with the femoral diaphysis, and the other following the midline of the body toward the axilla (Darrah et al., 2014; Bekteshi et al., 2021). The average of two consecutive measurements, with a 30-s rest break, was used for further analysis (Darrah et al., 2014; Johansen et al., 2020; Bekteshi et al., 2021).

Finally, participants were told to rate their self-reported pain intensity, using a Visual Analog Scale (VAS) (Declerck et al., 2016; Shearer et al., 2023), during the SLR test. With participants lying supine, the test was performed bilaterally. The leg was raised straight (knee extended and stretch position) at a speed of approximately 5°/s (Marsico et al., 2016b). Participants were instructed to inform the therapist once they reached the maximum tolerable symptoms. At that point, they were instructed to report their pain intensity. The therapist also registered the child’s description, and location of pain, tension, or tingling sensation. A first trial was conducted to allow familiarization with the produce, followed by two consecutive tests on each lower limb, with a 60-s rest between trials. The average of the two tests was used for further analysis (Kingsnorth et al., 2015; Barney et al., 2018; Shearer et al., 2023).

### Sample size

The sample size was estimated using G*Power software (version 3.1.9.2, University of Kiel, Kiel, Alemania). Based on a similar study in adult neurological patients (García-Bernal et al., 2022), we assumed an α level of 0.05, a statistical power of 80%, and a large effect size (η^2^ = 0.15) for differences between groups in muscle tone and stiffness using the myotonometer. As a result, a total of 40 participants (20 per group) was required.

### Statistical analysis

Statistical processing of the data was performed using SPSS Statistics for Windows software (version 29.0.2.0., IBM Corp, Armonk, NY). Quantitative variables are expressed as mean ± standard deviation (SD), while qualitative variables are presented as frequencies (percentages). Data normality was assessed using the Shapiro-Wilk test. A generalized linear mixed model (GLMM) was applied to detect differences in myotonometry (muscle tone and stiffness) and ROM and pain perception measurements, with sides (left vs. right) and sex (female vs. male) as within-subject factors, and groups (CP vs. control) as the between-subject factor. Bonferroni adjustment for *post hoc* multiple comparisons was used. In participants with CP, the Pearson product-moment correlation coefficient analysis with Bonferroni’s correction was used to explore possible associations between clinical features (CP type, affected side, medication use, and GMFCS level), and muscle tone and stiffness. Statistical significance was set at p < 0.05.

## Results

**FIGURE 1:**
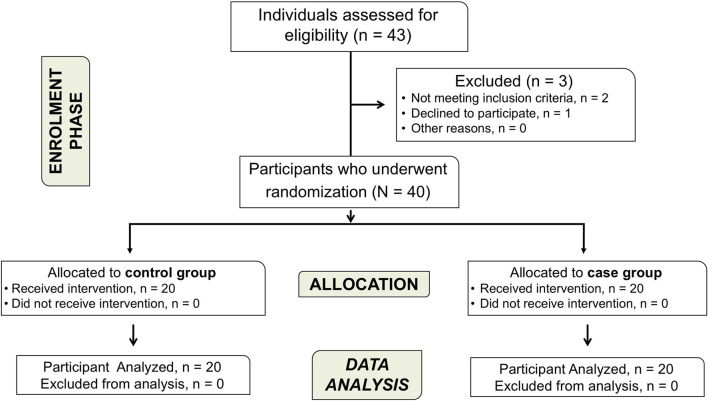
Flowchart diagram of participants.

A total of 40 participants (55% females, 22/40), with a mean age of 8.97 ± 1.74 years, agreed to participate and completed the study. The sample included 20 children with CP and 20 age- and sex-matched controls. No adverse reactions or dropouts occurred during the assessment protocol (Figure 1). Among children with CP, tetraparesis (9/20, 45%), and hemiparesis (8/20, 40%) were the most common presentations, with a predominantly bilateral involvement in more than half of participating minors (11/20, 55%). Participants were classified into GMFCS level I (5/20, 25%), level II (5/20, 25%), or level III (10/20, 50%). Both groups exhibited similar demographic characteristics, except for height (Table 1).

**TABLE 1:** Baseline clinical and demographic features of the study sample.

Study measures	Cerebral palsy group (n = 20)	Control group (n = 20)	*P* value
Age (yrs)	8.90 ± 1.88	9.05 ± 1.63	0.794^a^
Sex, female, % (n)	55% (11)	55% (11)	1.000^a^
Height, meters	1.28 ± 0.09	1.37 ± 0.13	0.008^a^
Weight, kilograms	32.02 ± 13.20	33.24 ± 8.95	0.565^a^
Body Mass Index (kg/cm^2^)	18.99 ± 5.72	17.19 ± 1.174	0.417^a^
CP type: tetraparesis, hemiparesis, diplegia, % (n)	45% (9); 40% (8); 15% (3)	N/A	
Affected side, left, right, bilateral, % (n)	20% (4); 25% (5); 55% (11)	N/A	
GMFCS, level I, level II, level III, % (n)	25% (5); 25% (5); 50% (10)	N/A	
Use of antispastic medication, yes, no, % (n)	20% (4); 80% (16)	N/A	

^a^ Mann-Whitney U test.

Data are expressed as mean ± standard deviation, or in frequencies (%).

Abbreviations: GMFCS, the gross motor function classification system.

### Muscle tone and stiffness

Table 2 presents the mean muscle stiffness (N/m) and tone (Hz) scores for the biceps femoris, rectus femoris, and adductor magnus (left and right sides) in both groups.

**TABLE 2:** Myotonometry measurements for muscle stiffness (N/m), and tone (Hz) in children with Cerebral Palsy (CP) and controls.

Location		Cerebral Palsy group (n = 20)		Control group (n = 20)	
	Measure	Left side	Right side	Left side	Right side
Hamstring (Biceps femoris)	Stiffness	196.80 ± 57.48	205.40 ± 66.78	190.35 ± 27.01	181.45 ± 35.53
	Tone	12.30 ± 1.95	12.91 ± 1.75	12.76 ± 0.93	12.64 ± 1.23
Quadriceps (Rectus femoris)	Stiffness	**204.44 ± 48.19** ^a^	**195.77 ± 45.36** ^a^	178.80 ± 26.94	175.65 ± 23.65
	Tone	12.42 ± 1.19	12.03 ± 1.07	12.06 ± 0.77	12.08 ± 0.60
Adductor	Stiffness	149.92 ± 42.62	**160.65 ± 50.35** ^a^	137.72 ± 22.27	139.37 ± 21.17
	Tone	10.79 ± 1.80	10.85 ± 1.30	10.90 ± 0.55	10.99 ± 0.48

^a^ Bold data indicates higher values in participants in the CP, group.

Data are expressed as mean ± standard deviation.

For muscle tone, the GLMM detected significantly higher values in males than females at the rectus femoris (F = 15.561; P < 0.001; η^2^ = 0.178) and adductor muscles (F = 8.579; P = 0.005; η^2^ = 0.106), with a significant group*sex interaction at both locations: rectus femoris: F = 5.599; P = 0.021; η^2^ = 0.072; adductor: F = 10.300; P = 0.002; η^2^ = 0.125. For muscle stiffness, significantly higher values were observed in the CP group at the rectus femoris (F = 9.042; P = 0.004; η^2^ = 0.112) and adductor muscles (F = 5.741; P = 0.019; η^2^ = 0.074). A significant sex effect was also found at the rectus femoris (F = 6.472; P = 0.013; η^2^ = 0.082), along with a group*sex interaction for the adductor muscle (F = 6.680; P = 0.012; η2 = 0.085).

### Lower limb range of motion and pain perception

Bilateral ROM measurements for hip flexion, knee extension, and ankle flexion in both groups are shown in Table 3. The GLMM demonstrated a significant group effect for hip flexion (F = 387.387; P < 0.001; η^2^ = 0.843), knee extension (F = 39.646; P < 0.001; η^2^ = 0.355), and ankle flexion (F = 1,684.244; P < 0.001; η^2^ = 0.959), with no sides or sex effect. Regarding pain perception (ROM during the SLR test), a significant group effect (F = 353.035; P < 0.001; η^2^ = 0.831), and group*sex interaction (F = 5.042; P = 0.028; η^2^ = 0.065) were observed.

**TABLE 3:** Range of motion for hip flexion, knee extension, and ankle flexion in children with Cerebral Palsy and controls.

Outcome measures	Cerebral Palsy group (n = 20)		Control group (n = 20)	
	Left side	Right side	Left side	Right side
SLR test	**54.55 ± 10.17** ^a^	**53.35 ± 11.43** ^a^	100.10 ± 12.40	99 ± 10.90
Hip flexion	**64.35 ± 12.30** ^a^	**65.55 ± 16.02** ^a^	119.60 ± 10.66	119.35 ± 8.94
Knee extension	**−14.40 ± 14.59** ^a^	**0.30 ± 0.92** ^a^	−16.90 ± 17.30	0.45 ± 1.09
Ankle flexion	**7.45 ± 5.57** ^a^	**76.75 ± 7.42** ^a^	6.35 ± 6.74	72.35 ± 8.57

^a^ Bold data indicates significant differences between groups.

Data are expressed as mean ± standard deviation.

Abbreviations: SLR, Straight leg raise test. Pain over 6 in a Numeric Pain Rating Scale (NPRS).

### Correlation analysis

Among participants with CP, significant correlations were identified between: a) the affected side and adductor muscle tone (r = 0.484, P = 0.030) and stiffness (r = 0.469, P = 0.037); b) and CP type and adductor muscle stiffness (r = 0.461, P = 0.041) (Table 4).

**TABLE 4:** Correlation coefficient matrix between clinical features of participants with CP and muscle tone and stiffness at hamstring, rectus femoris, and adductor muscles.

	CP type	Affected side	GMFCS	Use of medication	HMT tone	HMT stiffness	RF tone	RF stiffness	Adductor tone	Adductor stiffness
CP type	1	_	_	_	_	_	_	_	_	_
Affected side	**0.770** ^a^	1	_	_	_	_	_	_	_	_
GMFCS	**0.681** ^a^	**0.744** ^a^	1	_	_	_	_	_	_	_
Use of medication	0.165	0.168	−0.118	1	_	_	_	_	_	_
HMT tone	0.193	0.189	0.151	0.260	1	_	_	_	_	_
HMT stiffness	0.183	0.194	0.151	0.260	**0.980** ^a^	1	_	_	_	_
RF tone	0.383	0.365	0.172	0.390	**0.582** ^a^	**0.586** ^a^	1	_	_	_
RF stiffness	0.396	0.432	0.294	0.369	**0.505** ^a^	**0.526** ^a^	**0.807** ^a^	1	_	_
Adductor tone	0.360	**0.484** ^a^	0.213	0.304	0.299	0.358	**0.566** ^a^	**0.562** ^a^	1	_
Adductor stiffness	**0.461** ^a^	**0.469** ^a^	0.262	0.217	0.282	0.333	**0.478** ^a^	**0.602** ^a^	**0.880** ^a^	1

^a^ Bold date indicates statistically significant associations.

Note: Muscle tone and stiffness measurements are reported as the mean score between right and left sides in all locations.

Abbreviations: CP: cerebral palsy; GMFCS: the gross motor function classification system; RF: rectus femoris muscle.

## Discussion

The purpose of this study was to analyze the differences in children with neurological disorders associated with spasticity (CP) versus children with typical development in terms of intrinsic muscle properties, such as muscle stiffness and tone, ROM, and pain perception.

When considering myotonometry assessments, we only observed significant differences between groups for muscle stiffness of the rectus femoris and adductor magnus, but not for the biceps femoris. These results support those of Upachit et al., which suggests changes in myofascial and viscoelastic muscle properties, with a significant increase in muscle stiffness at rest, as well as mild muscle metabolism and atrophy, in individuals with pathologies associated with central degeneration (Upachit et al., 2025). In line with our results, Sagerer et al. reported increased stiffness and a short relaxation time in patients with neuromuscular disorders, highlighting the importance of intrinsic muscle tone and variability in tone distribution (Sagerer et al., 2024). Studies conducted in patients with stroke and Parkinson’s disease have also observed significant differences in stiffness when compared to healthy individuals (Brandín-de la Cruz et al., 2022; García-Bernal et al., 2022; 2023). In contrast to our results, Lukas et al. did not find significant differences in five disease groups regarding myotonometry assessments when comparing healthy individuals and patients with neuromuscular diseases. Measurements with the myotonometry device were unable to distinguish between the five different groups of disorders displaying increased stiffness or decreased muscle tone due to muscle atrophy (Lukas et al., 2023). For muscle tone (tension), as measured by myotonometry, there were no significant differences between the study groups. This may be due to the fact that both spasticity and hypertonia have a strong direct relationship with altered muscle tone, and sometimes this can create some confusion when identifying them (Hidler and Rymer, 1999). Interestingly, our findings suggest that sex seems to influence myotonometry values, with higher muscle tone and stiffness in males than in females, regardless of the study group, but only for the rectus femoris and adductor muscles. In line with this, Więch et al. (2020), investigated the effect of muscle tone on the body composition of children with CP, and reported a direct association between increased caloric needs and the increase of muscle tone, with differences between males and females in their study. As for correlations, significant associations were only found between adductor muscles tone or stiffness and some clinical parameters, such as CP type and the affected side. However, these results have to be taken cautiously, due to the small sample size and the observational design, which does not allow to imply causality. A rigorous assessment of potential sex differences should be addressed in future research involving a sufficiently large study sample.

As regards lower limb ROM, our findings showed significant differences between children with spasticity and normally developing peers. Reduced ROM and spasticity are frequent accompanying symptoms in CP that affect gait, posture, and daily function (Nordmark et al., 2009; Marsico et al., 2016a). Spasticity can also limit active joint ROM, and gross and fine motor function, with a potential influence to increase pain (Lindén et al., 2019). In the study by Nordmark et al. (2009), a decrease in the average ROM was found in children from 2 to 14 years in most assessed joints and muscles, especially for hip abduction and external rotation, knee extension, and ankle dorsiflexion. Cloodt et al. (2024) also concluded that the hamstrings muscles length decreased from childhood to adulthood in individuals with CP, regardless of the GMFCS level, and that knee extension was mainly reduced during the follow-up period for those with a GMFCS level between II to V. This supports previous results in children with CP, aged 4–17 years, who were able to walk at least 10 m on a flat, straight path (with or without assistive devices), and had significantly reduced passive ROM compared to children of the same age with typical development (McDowell et al., 2012).

Finally, regarding pain perception during the SLR test, the present results demonstrate significant differences between the study groups. The clinical measurement of hamstring muscles length is often used in decision-making regarding the evaluation and treatment of children with CP (Cloodt et al., 2021). In this regard, Marsico et al. (2016b) showed how children with spasticity report a higher degree of limitation in the SLR and suggested that pain perception prevented children to achieve a larger ROM while performing the test. Several authors have found that increased pain is more frequently localized in the lower limbs (between 32% and 82% of cases) and to a lesser extent in the upper limbs (4%–19% of cases) (Doralp and Bartlett, 2010; Mckinnon et al., 2019), which severely limits physical function (Certanec Gonzalez and Icarte Barrientos, 2017). In fact, one in four children and youth with CP experience moderate to severe pain that restricts daily life activities, with hip subluxation/dislocation and dystonia being the most common causes of this limitation. Additionally, nearly 4% of children/youth with CP complain of severe pain that prevents most movement-related activities, which has a profound social and emotional burden, especially during the early ages (Penner et al., 2013).

From a clinical perspective, our findings highlight the importance of targeted, muscle-specific interventions for children with CP, particularly focusing on reducing stiffness in the rectus femoris and adductor muscles. Objective biomechanical measures, such as myotonometry, may facilitate early identification of stiffness, guiding individualized treatments. Addressing reduced ROM and increased pain can significantly improve functional outcomes, mobility, and quality of life.

### Study limitations

As for the limitations of this study, we must mention that, despite the sample size estimation, the number of participants was not large enough to extrapolate the results. Also, participants with GMFCS level V were excluded, so it would be interesting to conduct the same study on a larger sample, including children and adolescents with GMFCS level V, in order to improve the generalizability of the findings. We did not collect data for the medication that children in the typically developing group may have been taking, which could potentially influence some of the current results. Despite the efforts to blind outcome assessors, due to the clinical characteristics of participants, an effective blinding was not feasible. Further studies should be developed to analyze and observe more accurately the intrinsic differences between spastic muscles and typical development muscles in children and adolescents.

## Conclusion

Our findings suggest differences in muscle stiffness, ROM, and pain perception during the SLR test, between children with CP and their typically developing peers, particularly highlighting greater stiffness in specific lower limb muscles. These findings emphasize the importance of objective biomechanical assessment to guide targeted interventions aimed at reducing muscle stiffness and improving functional outcomes. Future research should explore longitudinal changes in muscle properties and investigate the effectiveness of specific therapeutic modalities informed by biomechanical measurements, thus further optimizing clinical management strategies for children with CP.

## Data Availability

The raw data supporting the conclusions of this article will be made available by the authors, without undue reservation.
